# Pressure-Dependent Yield Stress of Organoclay-Based Gels

**DOI:** 10.1021/acs.langmuir.5c02052

**Published:** 2025-07-29

**Authors:** Nikolaos A. Burger, Benoit Loppinet, Andrew Clarke, George Petekidis

**Affiliations:** † IESL-FORTH, P.O. Box 1527, GR-711 10 Heraklion, Greece; ‡ Department of Materials Science & Engineering, University of Crete, Heraklion 70013, Greece; § SLB Cambridge Research, High Cross, Madingley Road, Cambridge CB3 0EL, U.K.

## Abstract

We provide a detailed investigation of the pressure and temperature dependence of flow curves and, in particular, of the yield stress, σ_y_, of a model drilling fluid formulation and the parent colloidal dispersion. We carefully considered the limitations of the high-pressure cell and designed reliable protocols covering a pressure range up to 100 MPa and a temperature range up to 85 °C. For both systems, the viscosity at high shear rates increased with pressure, scaling well with the pressure dependence of the solvent viscosity. For clay dispersions, σ_y_ increases slightly with temperature at a given pressure and much more with pressure across the whole temperature range (25–85 °C). For the drilling fluid formulation, σ_y_ increases with pressure and decreases with temperature. The time evolution of σ_y_ at high pressures (aging) was found to be different for the two systems. The clay dispersions did not show any aging of σ_y_, whereas the drilling fluid formulation shows a pressure-dependent aging of σ_y_. The aging is well captured by phenomenological kinetic models, with the amplitude of the aging being dependent on pressure but the kinetics not being dependent on pressure. The results suggest that the pressure does not affect much the volume fraction but rather affects the interactions between clay particles and emulsion droplets in the drilling fluid formulation. These new findings are in line with our previous results on the microscopic dynamics of the drilling fluid under high pressure and provide insights into the origin of the yield stress and its evolution with pressure and temperature in these technologically relevant systems.

## Graphical abstract

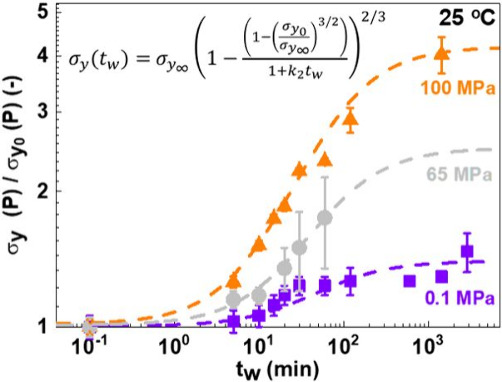

## Introduction

Appropriate drilling fluids are a key engineered component of the drilling process that have to meet safety, cost, and minimized environmental impact requirements while maintaining defined rheological properties and thermal stability.^1,2^ Functions include cooling the drill bit, carrying the cuttings to surface, maintaining wellbore stability, and controlling the wellbore pressure.^3–11^ Formulation of drilling fluids remains challenging.^12–14^ Although products are formulated compositions, the oil/water ratio, density, and viscosity are locally adjusted to account for the detailed design of any particular well. Oil-based, i.e., oil continuous, drilling fluids (OBF) are used routinely in the oil and gas industry.^8,15^ They are based on multicomponent colloidal dispersions and include surfactants for optimized clay dispersion and water emulsification, colloidal anisotropic particles as viscosity modifiers, and often barite as a weighting agent.^16–18^ The anisotropic particles are organophilic modified clays, (organoclays) that predominantly set the mechanical properties of full drilling fluid formulations.^16,19,20^ Depending on the morphology and size of the primary clay particles, dispersions at low-volume fractions are expected to form attractive colloidal gels.^21,22^ A common clay particle source is montmorillonite platelet nanoparticles.^23–27^ The exact structural type of the dispersions is rarely well-established but could be fractal-like (cf. laponite). The “aggregates” of primary nanoplatelet particles, often termed tactoids, have also been reported to be the building blocks for the dispersion.^26,28–33^ Studies have revealed that the surfactant also plays a role in the final properties of the organoclays and of the drilling fluid.^23,34,35^ Temperature–pressure treatment and clay volume fraction and water content also influence the structure and rheology of the dispersions.^36–38^

The mechanical properties (linear viscoelasticity, yield stress, and aging kinetics) and stability within extreme environments of a drilling fluid (and the associated clay particles) are vital requirements in application. Many studies report the mechanical behavior of organoclay-based dispersions with temperature, clay concentration, and surfactant type,^2,9,34,36,39^ but only a limited number are dedicated to the effect of pressure.^13,40^ The lack of high-pressure studies on gel properties is mostly due to experimental challenges, as rheological characterization under high pressure remains a difficult task. Capillary rheology or falling ball viscometry (for Newtonian suspensions) can be used under high pressures (100 or 700 MPa, respectively) but is limited to pressure drop and viscosity measurements, respectively.^41,42^ Industrial rheometers that are routinely used for measurements at HP-HT (200 MPa and 300 °C) are limited to six distinct shear rates of 3, 6, 100, 200, 300, and 600 rpm, need large sample volumes (hundreds of milliliters), and have limited sensitivity.^43^ On the other hand, recently proposed high-pressure passive microrheology imposes very restrictive optical properties on the systems and moreover can probe only linear viscoelasticity.^13,42,44–47^ Dedicated high-pressure shear cells are available to fit commercial rheometers and operate at up to 100 MPa. They make use of magnetic coupling to separate the ambient pressure of the main instrument and the high pressure of the sample cell. This coupling imposes significant limitations compared to that of a standard lab rheometer.^48,49^ In this work, we explore the high-pressure properties of two systems relevant to oil-based drilling fluids: a model formulation consisting of colloidal organoclay and brine in an oil emulsion and the colloidal dispersion only in the mineral oil. We use a commercial high-pressure rheological cell to evaluate the rheological properties through flow curves, covering a large range of pressures and temperatures. To evaluate the possible changes under pressure, we measured the isothermal compressibility and mineral oil viscosity as a function of temperature and pressure. We report the effect of sustained high pressure on the flow curves and especially on the yield stress and its aging.

## Materials and Methods

### Organoclay Particles

In this study, we used a hydrophobically modified (amine-treated) bentonite clay (VG-69 (a mark of SLB)) composed of platelets. Clay particles have a refractive index RI_clay_ ≈ 1.51, while the oil solvent has *R*_oil_ ≈ 1.447, at 25 °C.^13,19^ All chemical additives used to formulate the OBF were supplied by M-I SWACO, an SLB company.

### Organoclay Dispersions

The dispersions were prepared by using the organoclay particles, as received. We first added the mineral oil, Clairsol 370 (Haltermann Carless), and the organoclay was gradually added while mixing with an industrial homogenizer (Silverson L4RT) at 6000 rpm for at least 20 min. During the mixing, we added Milli-Q water (5 wt % of the dispersion clay content), which “activated” the organoclay by providing the necessary attraction (Scheme 1). Although the samples were prepared at room temperature (20 °C), during the high-shear procedure, the temperature was allowed to increase to about 45 °C. To ensure homogeneous dispersion without air bubbles prior to measurement, samples were placed overnight on a bottle roller.

**Scheme 1:**
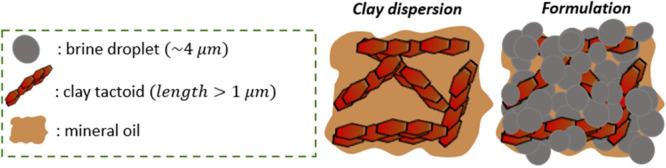
Schematic Diagram of the Two Systems Studied^a^

a Cartoons (not to scale) show tactoid clay (red platelets), mineral oil (background), brine droplets (gray circles), and the equivalent dispersions.

### Model Oil Continuous Drilling Fluid (VV1)

We chose a sample formulation, which was recently studied using diffusive wave spectroscopy (DWS).^13^ Our formulation comprised a 3 M (CaCl_2_) brine emulsion in Clairsol 370 mineral oil with clay particles together with sufficient Versamul versatile emulsifier package and Versacoat HF dispersant to ensure a stable system. The emulsion was 50% by volume of internal (brine) phase, which was emulsified by high-shear mixing at 6000 rpm using a Silverson mixer (L4RT) fitted with a square hole high-shear screen. The resulting droplet size was stable over time and approximately 4 μm in diameter, although of course polydisperse (Scheme 1).^13^

### Passive Microrheology

A continuous wave Nd:Yag laser (532 nm) was used, and the orientation of the glass windows enabled measurement at a θ = 90° scattering angle. A monomode optical fiber was used to couple the scattered light into a photomultiplier tube (PMT). The solution was pressurized by means of nitrogen gas, and the experiments were performed before the N_2_ molecules were expected to significantly diffuse in the sample.^45,46^ For high-pressure microviscometry experiments, poly­(methyl methacrylate) (PMMA) particles were added at a volume fraction of about 10^–4^ to act as probes.^45,50,51^ The particles, chemically grafted with poly­(hydroxystearic acid) (PHSA) chains (about 10 nm long) to ensure proper dispersion, had a total hydrodynamic radius of *R*_H_ = 130 nm (with a polydispersity on the order of 10%).^52^

### Isothermal Compressibility β_Τ_

Compressibility measurements were performed using a sealed ISCO D100 positive displacement pump in pressure control mode. Approximately 100 mL of sample was loaded into the barrel of the pump, and the end-cap assembled. Residual air in the cap was excluded by a small displacement, and the cap valve was closed. In pressure control mode, the pressure was increased stepwise with a flow rate limited to 2 mL/min. At each step, the volume of the system was recorded. The pressure–volume data were then used to calculate compressibility β_Τ_ at 25 °C, in pressure steps of 5 MPa, according to the equation $\beta_{Τ}=-\frac{1}{V}\frac{\partial V}{\partial P}{|}_{T}$

### Steady Shear Rheology at 0.1 MPa

For comparison with the high-pressure shear cell measurements, steady shear rheology was performed by using a conventional geometry at 0.1 MPa with a Malvern Kinexus Pro (NETZSCH) rotational rheometer. To reduce evaporation, all measurements were performed using a smooth Couette geometry with an internal cylinder diameter of 25 mm, a height of 37.5 mm, and a cup diameter of 27.5 mm. A comparison of the high-pressure cell with a standard rheometer configuration (Malvern) is represented in Figure S1B. The steady shear measurements of the formulation were performed at ambient pressure (0.1 MPa) and 25 °C. Good agreement was observed between the two cells. Minor effects of the geometry were expected for this shear strain rate regime. At the shear rates investigated in this study (γ̇ ≥ 1 s^–1^), wall slip was not expected in such systems, even with smooth geometry.^18^

### High-Pressure Rheometry

High-pressure rheometry tests were performed on a rotational stress-controlled rheometer (MCR 702, Anton Paar Co., Austria). The rheometer was used with a C-ETD 300/PR 1000 pressure cell and a CC29 bob concentric cylinder (Figure 1). The bob diameter was 29.5 mm with a height of the Couette area of 40 mm. The pressure cell’s internal diameter was 30 mm. The Couette geometry had an inner roughened surface and an outer smooth surface. The cell was pressurized with a Teleyne ISCO D65 positive displacement pump operating in pressure control mode. The applied pressure was verified and recorded with a second independent pressure gauge. The measurement cell employed a magnetic coupling that transfers the torque from the motor drive to the internal bob mounted inside the pressure cup with sapphire bearings at the extremities.

**Figure 1:**
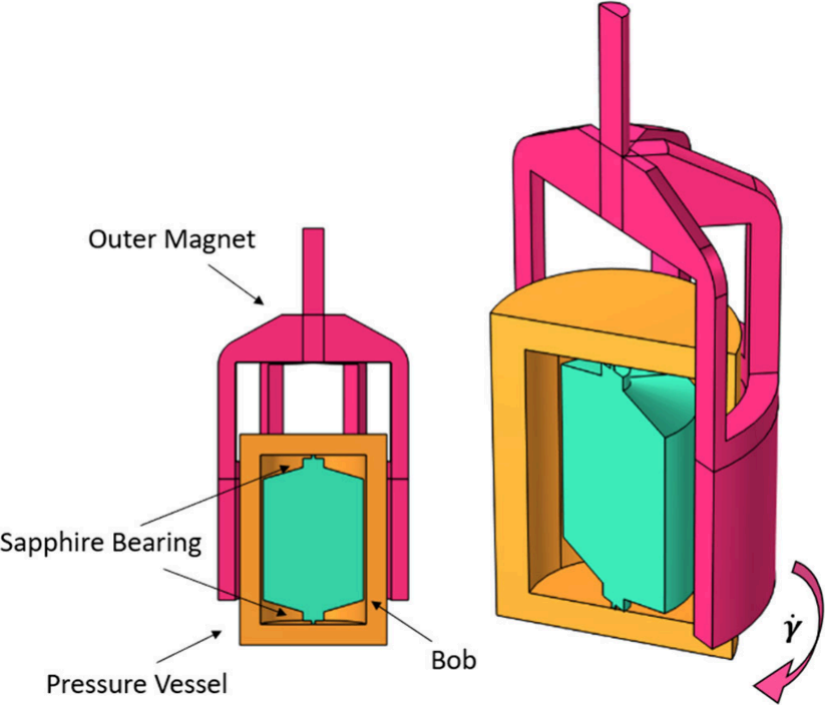
Cartoon showing the high-pressure vessel (yellow), bob (turquoise), and outer magnet (pink) stationary and during rotation, indicating the moving and stationary parts.

### High-Pressure Oscillatory Shear Rheology Limitation

High-pressure rheology studies remain rare, and a clear discussion on the limitation of the experimental setup is not superfluous in view of published works.

The most significant challenge in high-pressure shear rheology is the low torque resolution limited by the friction of the sapphire bearings within the cell, as compared to air bearings of lab rheometers for ambient-pressure measurements.^37,38,53^ System specifications give a minimum torque sensitivity of 50 μNm, which, however, after an air-check calibration, is found to be as low as about 15 μNm. Hence the minimum stress measurement is approximately 0.3 Pa. Thus, for oscillatory shear measurements with a 3% strain amplitude, a minimum measurable modulus is 10 Pa (Figure S1). In addition, the external magnetic coupler has a rather large radius and mass due to the embedded magnets, resulting in a large moment of inertia (*I* ≈ 0.08 kg m^2^). Thus, dynamic measurements are severely limited in frequency. More subtly, there is compliance of the magnetic coupling between the outer magnets and the magnets embedded in the bob. Hence, even a stiff solid will appear as a material with a finite modulus of about 300 Pa; i.e., if the bob is fixed, the compliance of the magnetic coupling will appear as a modulus. This also means that if the static friction of the bearings is such that the bob is effectively stuck at low strain amplitudes, then this compliance modulus will be seen. These limitations mean that dynamic and transient measurements, i.e., SAOS and step-strain or step-stress, are inaccessible for our materials. Hence, we restrict ourselves to steady-state measurements such as flow curves and examine the yield stress and aging kinetics at different pressures and temperatures.

It should also be noted that the investigation of pressure-induced hysteretic effects is hindered by the strain induced on any structure as pressure is released, which is due to the compressibility of the continuous phase. Given the cell dimensions and the oil compressibility (Figure 2), we estimate that a strain of approximately 25% is induced on reducing the pressure from 100 to 0.1 MPa, enough to disrupt any gel structure present.

**Figure 2:**
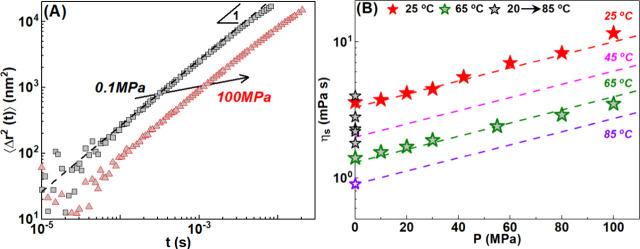
(A) Mean square displacement, ⟨Δ*r* ^2^(*t*)⟩, extracted from passive microrheology measurements in mineral oil at 0.1 MPa (black squares) and 100 MPa (red circles) at 25 °C. (B) Clairsol 370 viscosities and η_s_ as a function of pressure at 25 °C (filled red stars) and 65 °C (empty green stars). Black stars are attributed to 20, 35, 40, 45, and 85 °C, from top to bottom, respectively. Dashed lines represent exponential-like growth fits of the data. At 45 and 85 °C, η_s_ values were extracted from extrapolation of the fits at high pressures.

## Results and Discussion

### Mineral Oil under Pressure: Viscosity and Compressibility Measurements

We first report the pressure change of viscosity and compressibility of the mineral oil (Clarisol 370). We used passive microrheology to measure the pressure and temperature effects on oil viscosity (η_s_). For this, we dispersed PMMA particles in the oil and measured particle dynamics by dynamic light scattering (DLS). The field autocorrelation function, $C\left(t\right)=\frac{\left\langle I\left(q,t\right)I\left(q,0\right)\right\rangle }{\left\langle {I\left(q,t\right)}^{2}\right\rangle }$ at 25 °C with ⟨*I*(*q*, *t*)⟩ being the average scattered intensity at a scattering wavevector *q*, was used to extract the particle mean square displacement in a pressure range between 0.1 and 100 MPa (see Figure S2). In Figure 2A, we show the evolution of the probe mean square displacement (MSD), ⟨Δ*r* ^2^(*t*)⟩ (square nanometers), at 0.1 MPa (black symbols) and 100 MPa (red symbols). The linear relation between ⟨Δ*r* ^2^(*t*)⟩ and *t* confirms the diffusive motion of the colloidal probes within the temperature and pressure range examined. The slowing of the dynamics as seen by a decrease in ⟨Δ*r* ^2^(*t*)⟩ with an increase in pressure reflects the increase in solvent viscosity. In Figure 2B, we plot the evolution of Clairsol 370 viscosity, η_s_, with pressure at 25 °C (filled red stars) and 65 °C (filled gray green stars). We observed a typical exponential growth of the solvent viscosity with pressure, described by the equation η_s_(*T*, *P*) = η_0_(*T*, *P* = 0.1 MPa) exp­(*aP*), where η_0_ is the solvent viscosity at 0.1 MPa and *a* is a constant according to the Barrhus equation.^54^ Such viscosity evolution with pressure is also reported for many other organic solvents.^45^ At 45 and 85 °C, η_s_(*T*, *P*) values were extracted from extrapolation of the fits at high pressures.

### Isothermal Compressibility β_Τ_

In Figure 3, we report the evolution of isothermal compressibility values with pressure, in the ranges of 0.1–100 MPa for Clairsol 370 (olive squares) and 0.1–40 MPa for H_2_O (blue triangles), 25 wt % CaCl_2_ (cyan triangles), and the formulation (orange circles). The mineral oil shows the higher compressibility with a stronger pressure dependence compared to the less compressible brine (25 wt % CaCl_2_). The compressibility of the formulation (comprising approximately 50:50% brine in oil by volume) lies between the mineral oil and brine, coincidentally close to that of pure water. The larger volume change of the oil compared to the brine with increasing pressure implies a (small) increase in the brine volume fraction as well as the clay volume fraction in both the pure clay dispersion and the formulation. More specifically, the volume change (from 0.1 to 100 MPa) for the mineral oil is estimated to be 6.5%, whereas for the less compressible brine, the volume change is only 1.2% (from 0.1 to 40 MPa).

**Figure 3:**
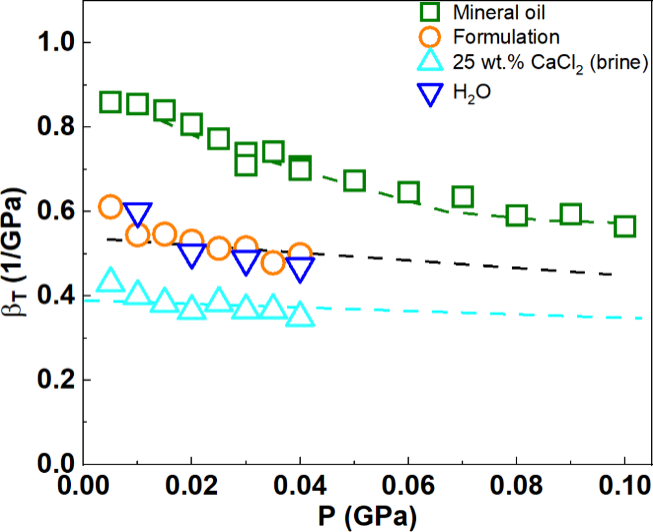
Evolution of compressibility β_Τ_ with pressure at 25 °C of H_2_O (empty blue triangles), mineral oil (empty olive squares), brine, 25 wt % CaCl_2_ (empty cyan triangles), and the formulation (empty orange circles).

### Flow Curve Protocols

We aimed for accurate measurements of flow curves in order to evaluate the yield stress, σ_y_, the infinite viscosity, η_∞_ (depicted in Figure 5), and their time evolution (aging). To do so, we used specific protocols. The shear protocols employed consist of four different steps: (i), a rejuvenation step to erase any shear history and ensure reproducibility between different measurements by shearing the dispersions at a shear rate γ̇ = 1000 s^–1^ for 60 s,^19^ (ii) steady shear sweeps from high to low shear rates (down flow curves) with 3 s per shear rate point and 10 points per decade resulting in an overall duration per flow curve of 90 s, (iii) aging at rest from 0 s to 48 h, and (iv) repeated flow curves from low to high shear rates (Table 1).

**Table 1:**
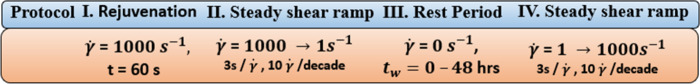
Rheological Protocol Followed to Probe Elastic (σ_y_) and Viscous (η) Contributions at Different Temperatures and Pressures with Waiting Times (*t*_w_) Varying from 0 s to 48 h

### Pressure Dependence of Flow Curves at 25 °C and *t*_w_ = 0

In Figure 4, we show flow curves (steady shear rate sweeps) for the clay dispersions and for the formulation at 0.1 MPa (squares), 65 MPa (circles), and 100 MPa (triangles) at 25 °C. Measurements were performed from high to low (filled symbols) and low to high (empty symbols) shear rates in order to detect possible hysteresis as discussed previously. With an increase in pressure from 0.1 to 100 MPa, we observed a gradual increase in stress with flow curves shifted almost vertically to higher values.

**Figure 4:**
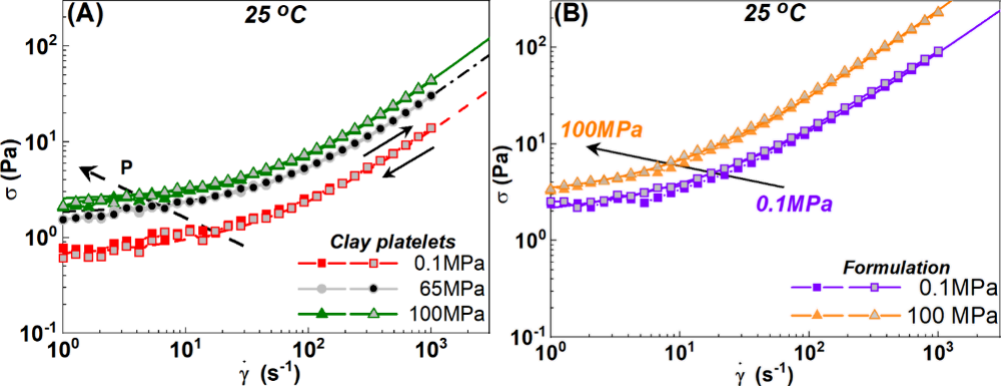
Stress as a function of shear rate at different pressures of (A) clay dispersions (5 wt %) at 0.1 MPa (red squares and triangles), 65 MPa (circles), and 100 MPa (triangles) and (B) the formulation at 0.1 MPa (red squares and triangles) and 100 MPa (triangles). Measurements performed from high to low (filled symbols) and low to high (gray filled symbols) shear rates. Lines represent TC fits of the experimental data.

For all temperatures and pressures studied, the dispersions demonstrate a typical yield stress response that is captured well by a Hershel–Buckley model (σ = σ_y_ + *k*γ̇^*n*^). It is equally well captured by the three-component (TC) model,^55^ as well, according to

1 $$\sigma =\sigma_{y}+\sigma_{y}{\left(\frac{\mathrm{\gamma ̇}}{{\mathrm{\gamma ̇}}_{c}}\right)}^{0.5}+\eta_{\mathrm{bg}}\mathrm{\gamma ̇}$$

where γ̇_c_ is the critical shear rate attributed to plastic contributions and η_bg_ is the background viscosity. Within the whole ranges of temperature (25–85 °C) and pressure (0.1–100 MPa) studied, no hysteresis is observed between the steady shear stress measured during a decreasing and an increasing shear rate sweep, indicating no structural difference between the two shear histories.

The same data can be plotted as the viscosity versus shear rate (Figure 5A, inset). We observe that the high shear viscosity at low and high pressure tends toward a constant value defining the steady-state infinite-shear viscosity, η_∞_. The steady-state value is captured well from the TC model with η_∞_ = η_bg_ (eq 1). As one can see in Figure 5, rescaling the data with pressure-dependent solvent viscosity η_s_ at the respective pressure (0.1 and 100 MPa) brings the two curves together. It reveals a minimal effect of pressure in the gel network for the clay dispersions. This suggests that the increase in stress with pressure comes mainly from the increased solvent viscosity. For the formulation (Figure 5B), the two curves do not superimpose, especially in the low-rate region. This suggests a weakening in the low-shear regime with an increase in pressure at 25 °C.

**Figure 5:**
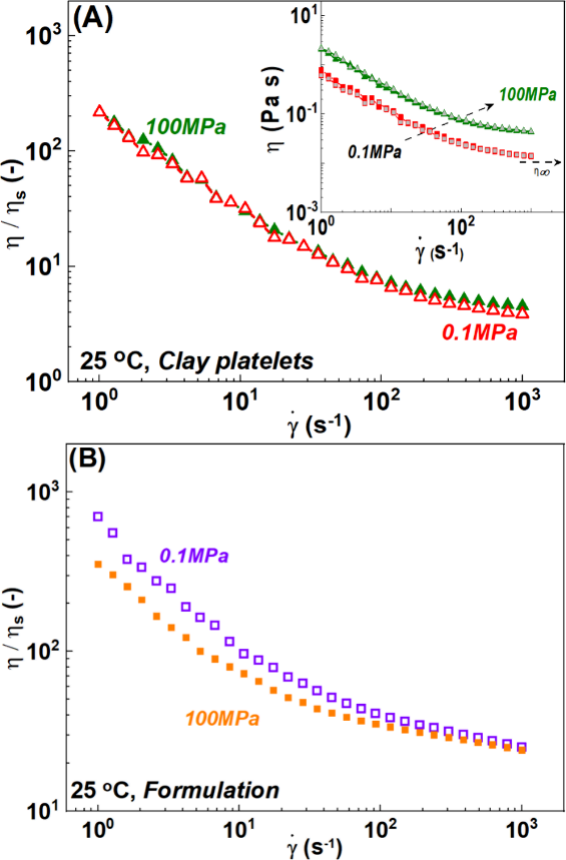
Flow curves (normalized viscosity with solvent viscosity as a function of shear rate γ̇) of (A) the clay dispersion (5 wt %) and (B) the formulation at 0.1 MPa (empty symbols) and 100 MPa (filled symbols) at 25 °C. For the sake of clarity, we show only data from high to low shear rates. The inset shows the flow curves (evolution of shear viscosity with shear rate) of clay dispersions (5 wt %) at 0.1 MPa (red squares and triangles) and 100 MPa (green triangles) at 25 °C. Measurements performed from high to low (filled symbols) and low to high (gray filled symbols) shear rates.

### Effect of Temperature on Flow Curves at Different Pressures at *t*_w_ = 0

In Figure 6, we show the equivalent steady shear rate sweeps (flow curves) for the formulation in the temperature range of 25–85 °C at three different pressures of 0.1 MPa (purple symbols), 65 MPa (black symbols), and 100 MPa (orange symbols) as indicated in the legends. Measurements were performed from high to low (filled symbols) and low to high (empty symbols) shear rates in order to detect possible hysteresis.^31,56–58^ At 0.1 MPa (Figure 6A), the stress decreases significantly with temperature, with both the elastic (yield stress σ_y_) and viscous (η_bg_) contributions becoming weaker (from purple squares at 25 °C to purple triangles at 85 °C). As we increase the pressure, we observe a weaker temperature dependence, and the flow curves at different temperatures are closer to each other (Figure 6B,C), suggesting that the increase in pressure stabilizes the nonlinear rheology of the formulation.

**Figure 6:**
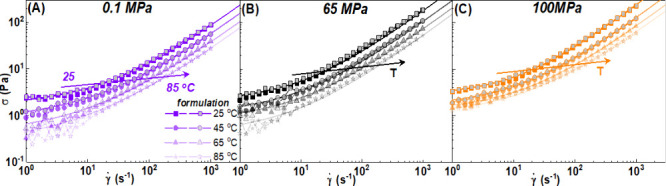
Flow curves for the formulation at 25 °C (squares), 45 °C (circles), 65 °C (triangles), and 85 °C (stars) at (A) 0.1 MPa (purple), (B) 65 MPa (black), and (C) 100 MPa (orange). Measurements performed from high to low (filled symbols) and low to high (empty gray symbols) shear rates. Lines represent TC fits of the experimental data.

In Figure 7A, we show a viscosity plot of the flow curves of the clay dispersions at different temperatures at room pressure. The viscosity (or the stress) increases with temperature in the low-shear rate regime where the elastic contributions are dominant and decreases in the high-shear rate regime where viscous contributions are dominant, as indicated by the black arrows. This is best represented in Figure 7B by rescaling the shear viscosity with solvent viscosity η_s_ at the respective temperature. This contradictory temperature behavior is the first indication that the network formation in the formulation (emulsion brine droplets trapped in an organoclay network) and clay dispersions is of a different nature, or that in the former (formulation) there are extra elastic contributions from the interactions of the clay particles and the emulsion droplets.^59,60^

**Figure 7:**
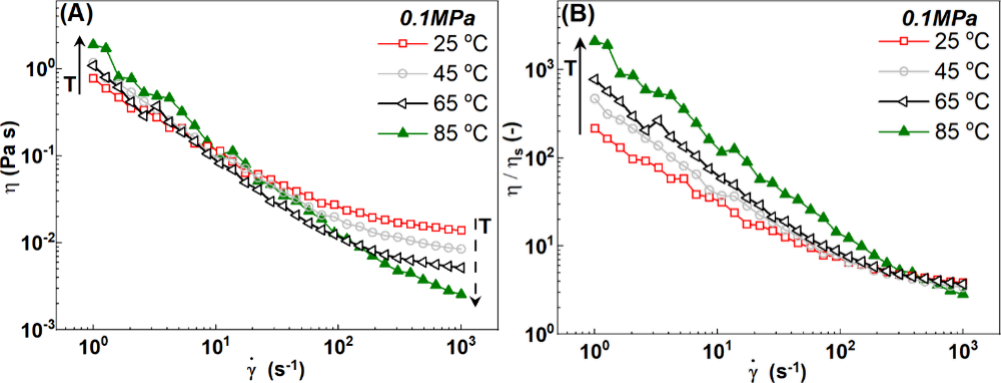
(A) Flow curves (evolution of shear viscosity with shear rate) of clay dispersions (5 wt %) at 25 °C (empty red squares), 45 °C (empty gray circles), 65 °C (empty black triangles), and 85 °C (filled green triangles) at 0.1 MPa. (B) Normalized viscosity with the solvent viscosity of the same dispersions at 0.1 MPa. For the sake of clarity, we show only data from high to low shear rates. The direction of the arrows in panels A and B indicates the increase in temperature from 25 to 85 °C. The stress evolution with temperature is shown in Figure S3.

Figure 8 sums up the pressure and temperature effects on yield stress σ_y_ for the clay dispersions (Figure 8A) and formulation (Figure 8B). The two samples display very different temperature behavior (as indicated by the arrows in Figure S4) but a similar response under pressure.

**Figure 8:**
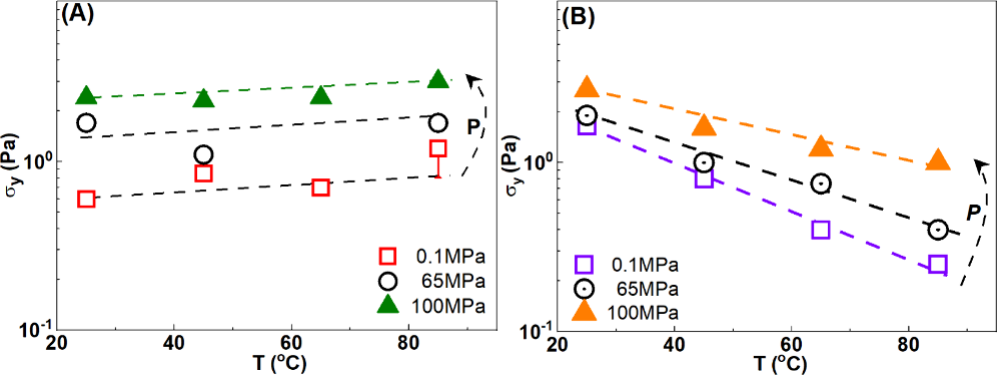
Yield stress σ_y_ for a nonaged sample (*t*_w_ = 0 s) as a function of temperature at 0.1 MPa (squares), 65 MPa (circles), and 100 MPa (triangles) of the (A) clay dispersion (5 wt %) and (B) formulation.

For clay dispersions, an increase in pressure leads to an increase in the elastic and viscous contributions in the whole temperature range and the yield stress, σ_y_, increases by a factor of 300% from 0.1 to100 MPa and increases slightly with temperature (from 25 to 85 °C). For the formulation, σ_y_ strengthens with pressure and weakens with temperature. At 100 MPa, the weaker temperature dependence of σ_y_ implies a lower activation energy (Figure S5); therefore, network formation is promoted at a high pressure. This observation holds only for *t*_w_ = 0. From the aging experiments at *t*_w_ = 60 min (described and discussed in the next section), we observe a rather similar temperature dependence of the yield stress at 0.1 and 100 MPa (Figure S6). This spectacular behavior of time- and temperature-dependent yield stress may have important industrial implications. Nevertheless, further studies of the yield stress evolution at longer waiting times and high temperatures and pressures are needed to draw robust conclusions. In the next section, we focus mostly on the time evolution of the yield stress at 25 °C and different pressures.

### *P* and *T* Dependence of High Shear Viscosity

At the highest measurable shear rate (γ̇ = 1000 s^–1^), the attractive interactions in clay dispersions and the formulation are overwhelmed by the strong shear forces so that the systems are expected to behave as dispersions of noninteracting particles.^29^ Figure 9 shows that the evolution of the infinite-shear viscosity with pressure is essentially driven by the pressure-dependent solvent viscosity as the ratio $\frac{\eta_{\infty }}{\eta_{s}}$ is almost independent of pressure and temperature.

**Figure 9:**
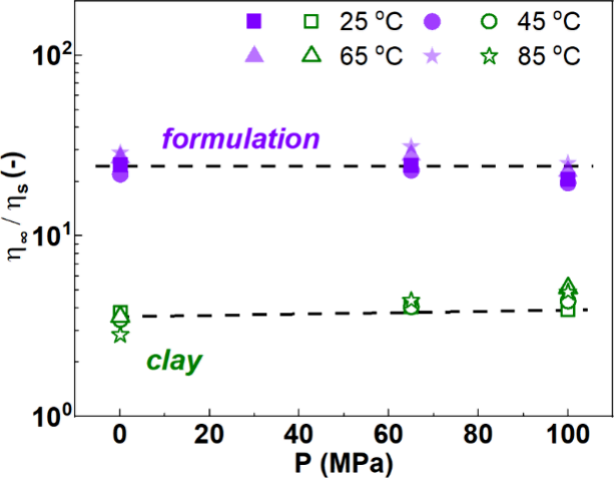
Ratio of the infinite shear viscosity to solvent viscosity, $\frac{\eta_{\infty }}{\eta_{s}}$ , with pressure of the clay dispersion (empty green symbols) and formulation (filled purple symbols) at 25 °C (squares), 45 °C (circles), 65 °C (triangles), and 85 °C (stars).

### Effect of Aging on Flow Curves

We next report the effects of increasing duration spent at a given pressure on the flow curves. For this purpose, we evaluate the evolution of the yield stress with waiting time at three different pressures of 0.1, 65, and 100 MPa over a range of temperatures. First, a rejuvenation (steady shear) is applied, followed by a decrease in shear rate from 1000 to 1 s^–1^. Then the dispersion is kept at rest for a certain waiting time, *t*_w_, and pressure before the flow curve measurement is performed using an increase in shear rate (from 1 to 1000 s^–1^) flow curve measurement.

In Figure 10, we report the flow curves obtained after different *t*_w_ (as indicated in the legends) at ambient pressure, 0.1 MPa (empty purple symbols), and high pressure, 100 MPa (filled orange symbols), at 25 °C. The series with the TC fits are shown in Figure S7. In Figure S8, we also report an effective yield stress defined as the stress value at the first point of each flow curve (at γ̇ = 1 s^–1^). The values of the effective yield (γ̇ = 1 s^–1^) are in very good agreement with the values derived from the TC model. They follow the same trend with a waiting time at any pressure. Deviations were observed only for long waiting times (Figure S8). To take into account the dependence of the determination methods, we considered as the yield stress the average value of the yield stress derived from both methods (γ̇ = 1 s^–1^ and the TC model fit). The difference between the two values is reported as the error in the σ_y_∞__/σ_y_0__ (−) in Table 2.

**Figure 10:**
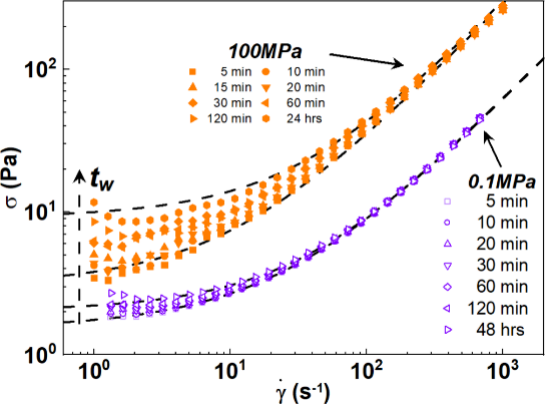
Flow curves of the formulation at 0.1 MPa (empty purple symbols) and 100 MPa (filled orange symbols). Measurements were performed from low to high shear rates after a specific period of waiting time at rest (*t*_w_) as indicated in the legends. Dashed lines represent TC fits of the experimental data. The black dashed arrow shows the increase in waiting time.

**Table 2:** Pressure Dependence of the Early-Time Yield Stress, σ_y_0__, Infinite-Time Yield Stress, σ_y_∞__, and σ_y_∞__/σ_y_0__ (−) Ratio Derived from (and in agreement with) eqs 2 and 3 ^a^

*P* (MPa)	σ_y_0__ (Pa)	σ_y_∞__ (Pa)	σ_y_∞__/σ_y_0__ (−)	*k*_1_ (min^–1^)	*k*_2_ (min^–1^)	β
0.1	1.7	2.4 ± 0.25	1.41 ± 0.2	0.024	0.016	0.75
65	2	4.5 ± 0.8	2.25 ± 0.4	0.022	0.016	0.75
100	2.6	11 ± 1	4.23 ± 0.4	0.024	0.016	0.75

a *k*_1_ and *k*_2_ are the recovery rate constants according to eqs 2 and 3, respectively.

The yield stress (as defined above) is plotted as a function of *t*_w_ at different pressures in Figure 11. There we see the time evolution (aging) of σ_y_ at 25 °C, for the formulation at all pressures measured (i.e., 0.1, 65, and 100 MPa). Aging of the yield stress is apparent in the formulation, with the amplitude increasing with pressure. Many efforts have been made to develop a rheological model of time-dependent yield stress aiming at microstructural insights through the structural recovery after shear cessation, related to the time evolution of the plateau modulus or yield stress.^57,61–65^ Such recovery is a common feature in aggregating attractive colloidal dispersions and gels. It is the basis of many thixotropic models. We fit our experimental yield stress data (Figure 11) with two simple thixotropic models, based on a first-order reaction kinetic process and a second-order kinetic process^66^ derived from Smoluchowski coagulation theory.^61,63,67^

**Figure 11:**
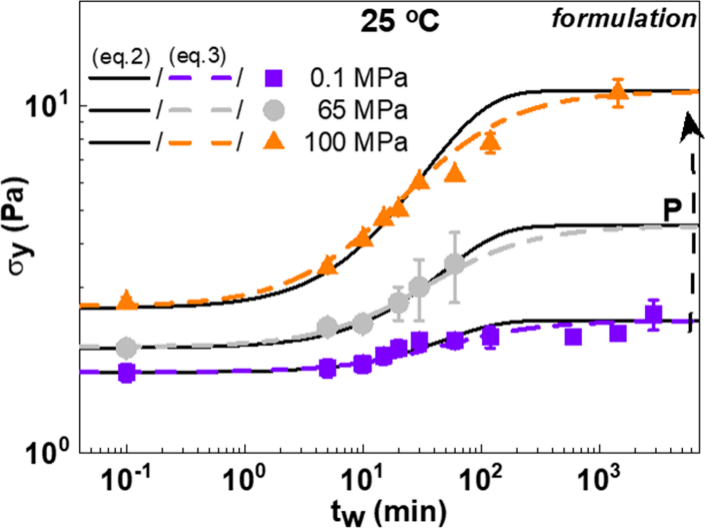
Yield stress σ_y_ as a function of waiting time *t*_w_ of the formulation at 25 °C and 0.1 MPa (purple squares), 65 MPa (gray circles), and 100 MPa (orange triangles), where the dashed lines are fits of the experimental data according to eq 2 ^61^ and the lines are fits to eq 3.^62^

In the first model,^68,69^ a structural parameter, ψ, representing a thixotropic structure and a rate equation for the description of time-dependent structural changes are used. The structural recovery after shear cessation is described by the kinetic equation $\frac{d\psi }{dt_{w}}=k_{1}\left(\psi^{E}-\psi \right)$ , where *k*_1_ is a recovery rate constant (with units of inverse time) and ψ^E^ reflects the recovered state at *t*_w_ → ∞. Assuming that the yield stress follows the same time evolution with the structural parameter, a first-order type of evolution with exponential decay is obtained, corresponding to a single recovery time. It did not fit the data very well, so we empirically introduced a stretch exponential function, with a stretch parameter β that would reflect a distribution of recovery times.

2 $$\sigma_{y}\left(t_{w}\right)=\sigma_{y_{\infty }}-\left(\sigma_{y_{\infty }}-\sigma_{y_{0}}\right)\;\exp{\left(-{k_{1}t}_{w}\right)}^{\beta }$$

where σ_y_0__ and σ_y_∞__ are the yield stress values at early and infinite times, respectively.

The second model we use is the Leong model,^63^ which is based on a second-order kinetic process^66^ and Smoluchowski coagulation theory^61,63,67^ (for more, see section IV in SI). It has been derived on the basis of the earlier model based on that of Hatori and Izuki^67^ where the mechanical properties (such as the viscosity) were assumed to be proportional to the concentration of the particle bonds. In that case, a time evolution of the yield stress, σ_y_(*t*_w_), was deduced as

3 $$\sigma_{y}\left(t_{w}\right)=\sigma_{y_{\infty }}{\left(1-\frac{1-{\left(\frac{\sigma_{y_{0}}}{\sigma_{y_{\infty }}}\right)}^{3/2}}{1+k_{2}t_{w}}\right)}^{2/3}$$

where σ_y_0__ and σ_y_∞__ are again the yield stress values at early and infinite times, respectively, and *k*_2_ is a recovery rate constant (similar to eq 2).

As one can see in Figure 11, both models capture well the aging time-dependent yield stress. The Leong model (eq 3) appeared to perform better at intermediate waiting times. The values of the fitting are listed in Table 2. The ratio $\frac{\sigma_{y_{\infty }}}{\sigma_{y_{0}}}$ increases from 1.41 at 0.1 MPa to 4.23 at 100 MPa. The recovery rate constants *k*_1_ and *k*_2_ derived from eqs 2 and 3, respectively, remain constant, independent of the pressure. Pressure seems to have only a minor effect on the recovery kinetic. The value of 0.75 for stretch exponential β is a reasonable number that does not imply a very broad distribution of recovery times.

We should note that other more elaborate thixotropy models^57,58,64,65^ may involve the evolution of both rheological quantities and internal microscopic relaxation times, aiming to provide further physical insights with respect to the origin of the observed macroscopic thixotropic response. However, here we probe only the time evolution of the yields stress. We refrain from using such models. This, however, might be the subject of future studies in which effects of aging on both the mechanical response (yield stress and/or viscoelastic moduli) and the internal dynamics will be followed.

We further note that in all samples, the aging was reversible through mechanical shear pointing toward some type of structural aging like the buildup of the structure and interconnected network formation.^13^ The yield stress of the clay dispersions does not change with the waiting time at any pressure (shown in Figure S11). This confirms that the aging observed in the formulation involves both the clay gel network and the emulsion droplets. At 85 °C, no aging of clay dispersions was observed over the whole pressure range; for the formulation, the flow curves are scattered and inconclusive (Figure S10).

### Discussion of the Effect of Pressure

Pressure has a strong effect on the yield stress of the clay dispersions (Figure 8), which increases by a factor of ∼3 from low pressure (0.1 MPa) to high pressure (100 MPa). One possible reason would be a change in volume fraction due to the difference in compressibility of the oil and the clay, given the facts that the yield stress evolves with clay concentration with the power law σ_y_ = *c* ^2.9^,^19^ and the increased volume fraction of the incompressible clay particles in the more compressible oil is estimated to be about 6.5%. The change in yield stress due to the change in volume fraction would be about ∼20%, far below the measured value of 300%. The effect of pressure on the yield stress rather originated in an enhancement of the interclay attractions with pressure. Several effects could cause such an increase, like the pressure-induced changes in the density, refractive index, and viscosity of the oil and the consequences for clay–clay interaction strength, or the stabilization of H-bonding by pressure.^45,46,70^

For the formulation, a 100 MPa increase in pressure results in an increase in yield stress of ∼200%. This is again much larger than the change expected from the volume fraction change. The pressure changes the volume fraction of the emulsion. Given the different compressibility of oil, β_Τ(oil)_ = 9.7 × 10^–10^ Pa^–1^, and brine, β_Τ(brine)_ = 4 × 10^–10^ Pa^–1^ (Figure 3), the emulsion volume fraction change is very limited from 0.5 to 0.516, the brine droplet volume fraction is estimated at 1% reduction in droplet size (at 100 MPa), and a 3.2% change in volume fraction of the clay particles in the continuous phase of the emulsion is noted. The rather small changes are not expected to account for the 200% change in yield stress.

Turning now to the aging behavior, we note that we did not observe any thixotropic behavior after flow cessation, in agreement with our recent study of organoclay dispersions at ambient pressures where we only observed a very slow evolution of the plateau modulus with waiting time.^19^

Pressure has a clear effect on the aging kinetics of the formulation (see Figures S8 and S9). The increased effects on aging yield stress suggest a slow structural rearrangement, more important at high pressures than at ambient pressure. The different behavior between the clay dispersion and the formulation suggests that the origin of the yield stress aging and its pressure dependence can be not only the change in interactions among the clay particles (i.e., the interactions in the gel network) but also the interactions between this clay gel network and the emulsion droplet. In agreement with our earlier DWS study,^13^ the formulation shows a strong pressure dependence on aging at room temperature. As discussed there, given the variation of the compressibility of the individual components, the sequence of refractive indices (calculated using the Lorentz–Lorentz approximation) for brine, oil, and clay will reorder, turning the brine–clay van der Waals interaction from repulsive at *P* < 70 MPa to weakly attractive at higher pressures (*P* > 70 MPa).^13^ Hence, a qualitative structural change between the sticky (at high pressure) emulsion droplets and the clay particles is expected at such pressures that would also be reflected in the yield stress evolution of the sample.

## Conclusions

Using a high-pressure cell on a commercial rheometer, we measured flow curves over a broad range of pressures for a model drilling fluid formulation comprised of an emulsion and clay particles. We established the pressure and temperature of yield stress σ_y_ and its aging under high pressure. We also observed pressure-dependent aging of the yield stress. The ratio of the aged yield stress to the rejuvenated yield stress at 0.1 MPa was 1.41, whereas at 100 MPa, it was 4.23. The aging kinetic (evolution from σ_y_0__ to σ_y_∞__) is captured well by simple kinetic models, and the characteristic time was essentially independent of pressure. The observed yield stress aging was always reversible through shear, supporting the hypothesis of the absence of permanent pressure-induced structural changes. At 85 °C, aging is weak at any pressure, which is also in line with previous findings at 65 °C.^13^ Our results are compatible with our previous findings on formulation dynamics.^13^ The clay only dispersion showed σ_y_ evolution different from that of the formulation, despite the fact that the organoclay network is expected to be at the origin of the yield stress of the formulation. We therefore conclude that interactions between the droplet emulsion and the clay gel network are at work in the formulation.

## Supplementary Material



## Notes

Oscillatory shear data and the reported limitations due to magnetic coupling, dynamic light scattering data of the solvent at different pressures, flow curves of clay dispersions at different pressures, yield stress evolution at different temperatures and pressures, Arrhenius-like representation of yield stress σ_y_ as a function of inverse temperature of the formulation at different pressures, flow curves and yield stress of the formulation and clay dispersions at different waiting times, pressures, and temperatures (PDF)

## References

[1] Li M.-C., Wu Q., Song K., Qing Y., Wu Y. (2015). Cellulose Nanoparticles
as Modifiers for Rheology and Fluid Loss in Bentonite Water-Based
Fluids. ACS Appl. Mater. Interfaces.

[2] Shi H., Jiang G., Shi H., Luo S. (2020). Study on Morphology
and Rheological Property of Organoclay Dispersions in Soybean Oil
Fatty Acid Ethyl Ester over a Wide Temperature Range. ACS Omega.

[3] Kosynkin D. V., Ceriotti G., Wilson K. C., Lomeda J. R., Scorsone J. T., Patel A. D., Friedheim J. E., Tour J. M. (2012). Graphene Oxide as
a High-Performance Fluid-Loss-Control Additive in Water-Based Drilling
Fluids. ACS Appl. Mater. Interfaces.

[4] Kumari W. G. P., Ranjith P. G. (2019). Sustainable Development
of Enhanced Geothermal Systems
Based on Geotechnical Research – A Review. Earth-Sci. Rev..

[5] Collins I. R., Cano Floriano D., Paevskiy I., Wee J., Boek E. S., Mohammadi M. K. (2024). Transition
from Oil & Gas Drilling Fluids to Geothermal
Drilling Fluids. Geoenergy Sci. Eng..

[6] Li D., Zhang D., Li F., Xiang Q., Dong Y., Wang L. (2024). Fundamental Studies
on Fluids-Independent Regenerative Nanocomposite
Hydrogels for Fracture Treatments of Conformance Control. ACS Appl. Mater. Interfaces.

[7] Wu B., Zhang X., Jeffrey R. G., Bunger A. P., Jia S. (2016). A Simplified
Model for Heat Extraction by Circulating Fluid through a Closed-Loop
Multiple-Fracture Enhanced Geothermal System. Appl. Energy.

[8] Mahmoud A., Gajbhiye R., Elkatatny S. (2023). Application
of Organoclays in Oil-Based
Drilling Fluids: A Review. ACS Omega.

[9] Li Q., de Viguerie L., Laporte L., Berraud-Pache R., Zhuang G., Souprayen C., Jaber M. (2024). Oily Bioorganoclays
in Drilling Fluids: Micro and Macroscopic Properties. Appl. Clay Sci..

[10] Huang X., Shen H., Sun J., Lv K., Liu J., Dong X., Luo S. (2018). Nanoscale Laponite as a Potential
Shale Inhibitor in Water-Based Drilling Fluid for Stabilization of
Wellbore Stability and Mechanism Study. ACS
Appl. Mater. Interfaces.

[11] Li M.-C., Wu Q., Song K., Lee S., Jin C., Ren S., Lei T. (2015). Soy Protein Isolate
As Fluid Loss Additive in Bentonite–Water-Based
Drilling Fluids. ACS Appl. Mater. Interfaces.

[12] Ali M., Jarni H. H., Aftab A., Ismail A. R., Saady N. M. C., Sahito M. F., Keshavarz A., Iglauer S., Sarmadivaleh M. (2020). Nanomaterial-Based
Drilling Fluids for Exploitation of Unconventional Reservoirs: A Review. Energies.

[13] Clarke A., Jamie E., Burger N. A., Loppinet B., Petekidis G. (2022). A Microstructural
Investigation of an Industrial Attractive Gel at Pressure and Temperature. Soft Matter.

[14] Li, Q. ; Zhuang, G. ; Yuan, P. ; Bergaya, F. Chapter 12 - Future Challenges Related to Clay Minerals in Drilling and Drilling Fluids. In Clay Science in Drilling and Drilling Fluids; Zhuang, G. , Yuan, P. , Eds.; Developments in Clay Science; Elsevier, 2024; Vol. 11, pp 313–338. 10.1016/B978-0-443-15598-7.00006-7

[15] Geng Y., Sun J., Wang J., Wang R., Yang J., Wang Q., Ni X. (2021). Modified Nanopolystyrene
as a Plugging Agent for Oil-Based Drilling
Fluids Applied in Shale Formation. Energy Fuels.

[16] Clarke A. (2021). Gel Breakdown
in a Formulated Product via Accumulated Strain. Soft Matter.

[17] Bergaya, F. ; Lagaly, G. Chapter 1 - General Introduction: Clays, Clay Minerals, and Clay Science. In Developments in Clay Science; Bergaya, F. , Lagaly, G. , Eds.; Handbook of Clay Science; Elsevier, 2013; Vol. 5, pp 1–19. 10.1016/B978-0-08-098258-8.00001-8

[18] Zhuang, G. ; Li, Q. ; Bergaya, F. ; Yuan, P. Chapter 1 - The Significance of Clay Minerals in Drilling and Drilling Fluids. In Clay Science in Drilling and Drilling Fluids; Zhuang, G. , Yuan, P. , Eds.; Developments in Clay Science; Elsevier, 2024; Vol. 11, pp 1–19. 10.1016/B978-0-443-15598-7.00003-1

[19] Burger N. A., Loppinet B., Clarke A., Petekidis G. (2024). Tuning the
Mechanical Properties of Organophilic Clay Dispersions: Particle Composition
and Preshear History Effects. J. Rheol..

[20] Suter J. L., Groen D., Coveney P. V. (2015). Mechanism
of Exfoliation and Prediction
of Materials Properties of Clay–Polymer Nanocomposites from
Multiscale Modeling. Nano Lett..

[21] Tester C. C., Aloni S., Gilbert B., Banfield J. F. (2016). Short- and Long-Range
Attractive Forces That Influence the Structure of Montmorillonite
Osmotic Hydrates. Langmuir.

[22] Ganley W. J., van Duijneveldt J. S. (2017). Controlling
the Rheology of Montmorillonite Stabilized
Oil-in-Water Emulsions. Langmuir.

[23] Stockmeyer M. R. (1991). Adsorption
of Organic Compounds on Organophilic Bentonites. Appl. Clay Sci..

[24] Lan Y., Liu Y., Li J., Chen D., He G., Parkin I. P. (2021). Natural
Clay-Based Materials for Energy Storage and Conversion Applications. Adv. Sci..

[25] Ahmad H. M., Murtaza M., Gbadamosi A., Kamal M. S., Hussain S. M. S., Mahmoud M., Patil S. (2023). Application
of Novel Magnetic Surfactant-Based
Drilling Fluids for Clay Swelling Inhibition. Energy Fuels.

[26] Burger N. A., Loppinet B., Clarke A., Petekidis G. (2025). How Preparation
Protocols Control the Rheology of Organoclay Gels. Ind. Eng. Chem. Res..

[27] Segad M., Jönsson B., Åkesson T., Cabane B. (2010). Ca/Na Montmorillonite:
Structure, Forces and Swelling Properties. Langmuir.

[28] King H. E., Milner S. T., Lin M. Y., Singh J. P., Mason T. G. (2007). Structure
and Rheology of Organoclay Suspensions. Phys.
Rev. E.

[29] Koumakis N., Moghimi E., Besseling R., Poon W. C. K., Brady J. F., Petekidis G. (2015). Tuning Colloidal
Gels by Shear. Soft Matter.

[30] Moghimi E., Jacob A. R., Koumakis N., Petekidis G. (2017). Colloidal
Gels Tuned by Oscillatory Shear. Soft Matter.

[31] Joshi Y. M., Petekidis G. (2018). Yield Stress
Fluids and Ageing. Rheol. Acta.

[32] Petekidis, G. ; Wagner, N. J. Rheology of Colloidal Glasses and Gels. In Theory and Applications of Colloidal Suspension Rheology; Mewis, J. , Wagner, N. J. , Eds.; Cambridge Series in Chemical Engineering; Cambridge University Press: Cambridge, U.K., 2021; pp 173–226. 10.1017/9781108394826.006

[33] Ganley W. J., van Duijneveldt J. S. (2015). Controlling
Clusters of Colloidal Platelets: Effects
of Edge and Face Surface Chemistries on the Behavior of Montmorillonite
Suspensions. Langmuir.

[34] Hedley C. B., Yuan G., Theng B. K. G. (2007). Thermal
Analysis of Montmorillonites
Modified with Quaternary Phosphonium and Ammonium Surfactants. Appl. Clay Sci..

[35] Zhuang G., Zhang Z., Peng S., Gao J., Pereira F. A. R., Jaber M. (2019). The Interaction between
Surfactants and Montmorillonite
and Its Influence on the Properties of Organo-Montmorillonite in Oil-Based
Drilling FluIDS. Clays Clay Miner.

[36] Zhuang G., Zhang Z., Jaber M. (2019). Organoclays
Used as Colloidal and
Rheological Additives in Oil-Based Drilling Fluids: An Overview. Appl. Clay Sci..

[37] Ahuja A., Lee R., Joshi Y. M. (2021). Advances and Challenges
in the High-Pressure Rheology
of Complex Fluids. Adv. Colloid Interface Sci..

[38] Münstedt H. (2020). Influence
of Hydrostatic Pressure on Rheological Properties of Polymer Melts—A
Review. J. Rheol..

[39] Fan Z., Zhang L., Liu S., Luan L., Li G., Sun D. (2019). Mechanism of High Temperature
Induced Destabilization of Nonpolar
Organoclay Suspension. J. Colloid Interface
Sci..

[40] Hermoso J., Martinez-Boza F., Gallegos C. (2015). Influence of Aqueous Phase Volume
Fraction, Organoclay Concentration and Pressure on Invert-Emulsion
Oil Muds Rheology. J. Ind. Eng. Chem..

[41] Cardinaels R., Van Puyvelde P., Moldenaers P. (2007). Evaluation and Comparison of Routes
to Obtain Pressure Coefficients from High-Pressure Capillary Rheometry
Data. Rheol. Acta.

[42] Gekko K., Fukamizu M. (1991). Effect of Pressure
on the Sol-Gel Transition of Gelatin. Int. J.
Biol. Macromol..

[43] Abdo J., Haneef M. D. (2013). Clay Nanoparticles Modified Drilling
Fluids for Drilling
of Deep Hydrocarbon Wells. Appl. Clay Sci..

[44] Dennis K. A., Gao Y., Phatak A., Sullivan P. F., Furst E. M. (2020). Design, Operation,
and Validation of a Microrheology Instrument for High-Pressure Linear
Viscoelasticity Measurements. J. Rheol..

[45] Burger N.
A., Meier G., Bouteiller L., Loppinet B., Vlassopoulos D. (2022). Dynamics and
Rheology of Supramolecular Assemblies at Elevated Pressures. J. Phys. Chem. B.

[46] Burger N. A., Mavromanolakis A., Meier G., Brocorens P., Lazzaroni R., Bouteiller L., Loppinet B., Vlassopoulos D. (2021). Stabilization
of Supramolecular Polymer Phase at High Pressures. ACS Macro Lett..

[47] Burger N. A., Meier G., Vlassopoulos D., Loppinet B. (2025). High-Pressure Effects
on Gelatin Sol–Gel Transition. Ind. Eng.
Chem. Res..

[48] Behzadfar E., Hatzikiriakos S. G. (2014). Rheology of Bitumen: Effects of Temperature,
Pressure,
CO2 Concentration and Shear Rate. Fuel.

[49] Behzadfar E., Hatzikiriakos S. G. (2014). Diffusivity
of CO2 in Bitumen: Pressure–Decay
Measurements Coupled with Rheometry. Energy
Fuels.

[50] Burger N. A., Pembouong G., Bouteiller L., Vlassopoulos D., Loppinet B. (2022). Complete Dynamic Phase Diagram of a Supramolecular
Polymer. Macromolecules.

[51] Vereroudakis E., Burger N. A., Bouteiller L., Loppinet B., Meijer E. W., Vlassopoulos D., Van Zee N. J. (2024). Counterintuitive Viscoelasticity
of Supramolecular Polymer Networks Driven by Coassembly with Water
Molecules. Macromolecules.

[52] Royall C. P., Poon W. C. K., Weeks E. R. (2013). In Search
of Colloidal Hard Spheres. Soft Matter.

[53] Kulisiewicz L., Delgado A. (2010). High-Pressure
Rheological Measurement Methods: A Review. Appl.
Rheol..

[54] Barus C. (1893). Isothermals,
Isopiestics and Isometrics Relative to Viscosity. Am. J. Sci..

[55] Caggioni M., Trappe V., Spicer P. T. (2020). Variations
of the Herschel–Bulkley
Exponent Reflecting Contributions of the Viscous Continuous Phase
to the Shear Rate-Dependent Stress of Soft Glassy Materials. J. Rheol..

[56] Rathinaraj J. D. J., Lennon K. R., Gonzalez M., Santra A., Swan J. W., McKinley G. H. (2023). Elastoviscoplasticity, Hyperaging, and Time–Age-Time–Temperature
Superposition in Aqueous Dispersions of Bentonite Clay. Soft Matter.

[57] Bhattacharyya T., Jacob A. R., Petekidis G., Joshi Y. M. (2023). On the Nature of
Flow Curve and Categorization of Thixotropic Yield Stress Materials. J. Rheol..

[58] Sharma S., Shankar V., Joshi Y. M. (2023). Viscoelasticity and Rheological Hysteresis. J. Rheol..

[59] Abbasian
Chaleshtari Z., Salimi-Kenari H., Foudazi R. (2021). Interdroplet Interactions
and Rheology of Concentrated Nanoemulsions for Templating Porous Polymers. Langmuir.

[60] Valipour
Goodarzi B., Foudazi R. (2025). Rheology and Structure of Emulsions. Langmuir.

[61] de
Kretser R. G., Boger D. V. (2001). A Structural Model for the Time-Dependent
Recovery of Mineral Suspensions. Rheol. Acta.

[62] Nguyen Q. D., Boger D. V. (1987). Characterization of Yield Stress Fluids with Concentric
Cylinder Viscometers. Rheol. Acta.

[63] Leong Y. K., Clode P. L. (2023). Time-Dependent Clay
Gels: Stepdown Shear Rate Behavior,
Microstructure, Ageing, and Phase State Ambiguity. Phys. Fluids.

[64] Joshi Y. M. (2024). PERSPECTIVE:
Analysis of Thixotropic Timescalea). J. Rheol..

[65] Joshi Y. M. (2025). Linear
Viscoelasticity of Physically Aging Soft Glassy (Thixotropic) Materials. Curr. Opin. Colloid Interface Sci..

[66] Mewis J. (1979). Thixotropy
- a General Review. J. Non-Newton. Fluid Mech..

[67] Hattori K., Izumi K. (1982). A Rheological Expression of Coagulation Rate Theory. J. Dispers. Sci. Technol..

[68] Lapasin R., Papo A., Rajgelj S. (1983). The Phenomenological
Description
of the Thixotropic Behaviour of Fresh Cement Pastes. Rheol. Acta.

[69] Nguyen Q. D., Boger D. V. (1985). Thixotropic Behaviour of Concentrated Bauxite Residue
Suspensions. Rheol. Acta.

[70] Dougherty R. C. (1998). Temperature
and Pressure Dependence of Hydrogen Bond Strength: A Perturbation
Molecular Orbital Approach. J. Chem. Phys..

