# Supplementary material for: Pressure-Dependent Yield Stress of Organoclay-Based Gels
Source: Langmuir. 2025 Jul 29;41(31):20638–47. doi: 10.1021/acs.langmuir.5c02052 (PMC12356070; doi:10.1021/acs.langmuir.5c02052)
Supplement: Supplementary file 1 [file la5c02052_si_001.pdf]

Supporting information

Pressure-dependent yield stress of organoclay-based gels

Nikolaos A. Burger[1,2*], Benoit Loppinet[1], Andrew Clarke[3*] and George Petekidis[1 2]

[1] IESL-FORTH, P.O. Box 1527, GR-711 10 Heraklion, Greece
[2]Department of Materials Science & Engineering, University of Crete, Heraklion 70013, Greece
[3]SLB Cambridge Research, High Cross, Madingley Road, Cambridge CB3 0EL, UK

burger_nik0s@hotmail.gr

AClarke3@slb.com

Content

-

I. Oscillatory shear data and the reported limitations due to magnetic coupling of HP cell.
II. Solvent viscosity at pressure.
III. Flow curve at pressure & temperature for aged gels, $t_w > 0$.
IV. Description of Leong Model for yield stress evolution.

I. Oscillatory shear data and the reported limitations due to magnetic coupling of HP cell.

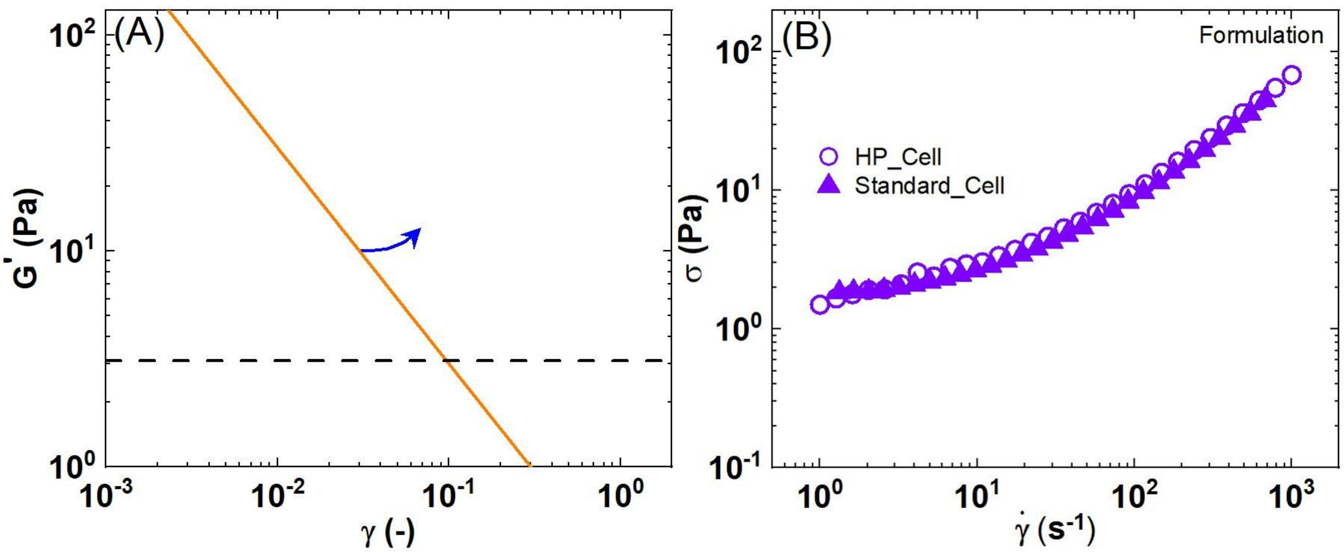

Figure S1. (A) Storage modulus, $G'(Pa)$ as a function of oscillatory strain amplitude ($\omega = 1\,Hz$). The orange and black dashed lines indicate the accessible area (top right) if we assume a minimum stress $0.3\,Pa$. For minimum stress of $1\,Pa$, the orange line is shifted horizontally to higher values. (B) Comparison between steady shear measurements performed using a standard rheometer configuration (filled purple triangles) and with the pressure cell (open purple circles) at P = 0.1 MPa and T = 25 °C. Further details are described in the Materials and Methods section.

II. Solvent viscosity at pressure

In a dynamic light scattering experiment the intensity correlation functions $g^{(2)}(q,\tau)$ delivered by the correlator is the correlated scattering intensity at two different periods of time, $t, t+\tau$ normalized with the square of the mean intensity. So, for ergodic samples, the intensity correlation function ranges from unit to zero amplitude.

$$g^{(2)}(q,\tau) = \frac{\langle I(q,0)\, I(q,\tau)\rangle}{\langle I(q)\rangle^2}$$

(S1)

Where the field correlation function, $g^{(1)}(q,\tau)$

$$g^{(1)}(q,\tau)=\frac{\langle E(q,t=0)E^{¿}(q,\tau)\rangle}{\langle I(q)\rangle}$$

(S2)

The field autocorrelation function (FAF), $g^{(1)}(q,\tau)\sim\ C(t)$ is obtained using the Siegert relation, applicable for ergodic signals,

$$g^{(1)}(q,\tau)=\sqrt{\frac{g^{(2)}(q,\tau)-1}{f^{¿}}}$$

(S3)

In case the scattered intensity arises from dilute colloidal tracers, the electric field autocorrelation function $g_1(t)$is related to the mean square displacement $\langle\Delta r^2\rangle$ of the colloidal probe particle by

$$g^{(1)}(q,\tau)=\exp\left(\frac{-q^2\langle\Delta r^2(\tau)\rangle}{6}\right)$$

(S4) $f^{¿}$ is an experimental factor (close to 1 in our case owing to the use of a mono-mode optical fiber). The scattering wave vector (q),$q=\frac{4\pi n}{\lambda}\sin\left(\frac{\theta}{2}\right)$, In this study, the refractive index ($n=1.49$), the wavelength of laser light ($\lambda=532\,nm$) and the scattering angle ($\theta=90°$). That returns q $¿\,0.024\,nm^{-1}$. For a viscous fluid (mineral oil), the Stokes-Einstein-Sutherland (SES) equation (5) below links the macroscopic (viscosity) and microscopic (thermal energy $k_B T$) properties. In particular, in a medium of viscosity η_s, the diffusion coefficient of the embedded probe with hydrodynamic radius R_h is

$$D=\frac{k_B T}{6\pi\eta_s R_h} \qquad \text{(S5)}$$

All $C(t)$ in Figure S2 are well fitted with a single exponential decay function, $C(t)=Ae^{-q^2Dt}$, with A the amplitude, q the scattering wave vector in nm^{-1} and D the diffusion coefficient in cm^2/s.

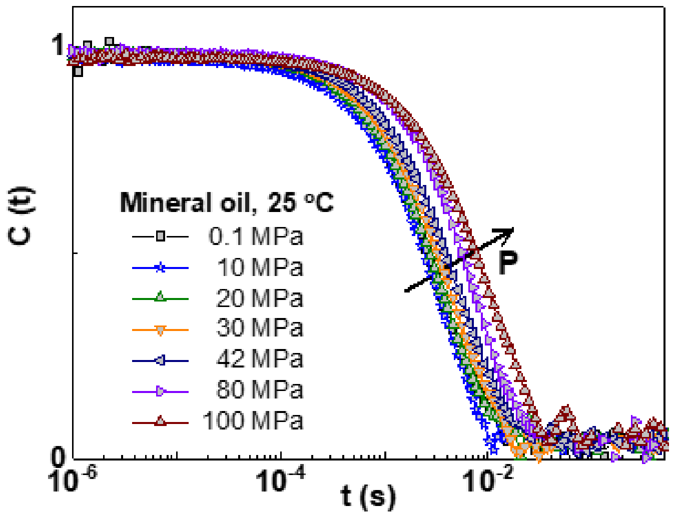

Figure S2. Field autocorrelation functions, $C(t)$, extracted from passive microrheology measurements in Clairsol 370. Legends indicate the equivalent pressure (MPa) at 25 ℃.

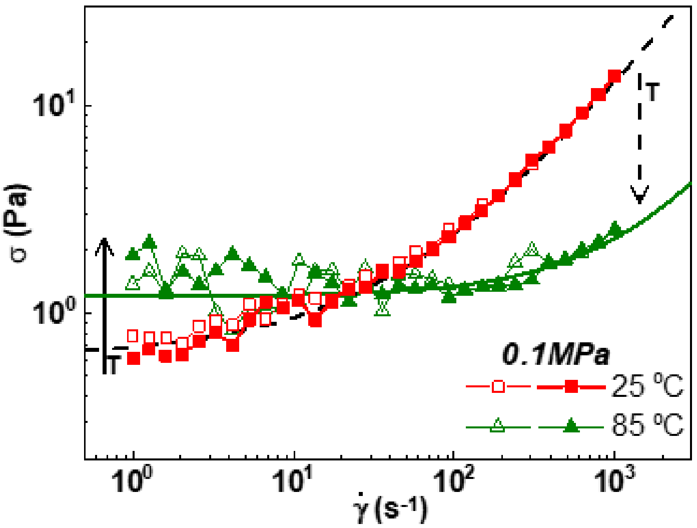

Figure S3. Flow curves of clay dispersions (5 wt. %) at 0.1 (red squares) and 100 MPa (green triangles) at 25 ℃. Measurements performed from high to low (filled) and low to high (grey filled symbols) shear rates. Dashed lines represent T-C fits of the experimental data.

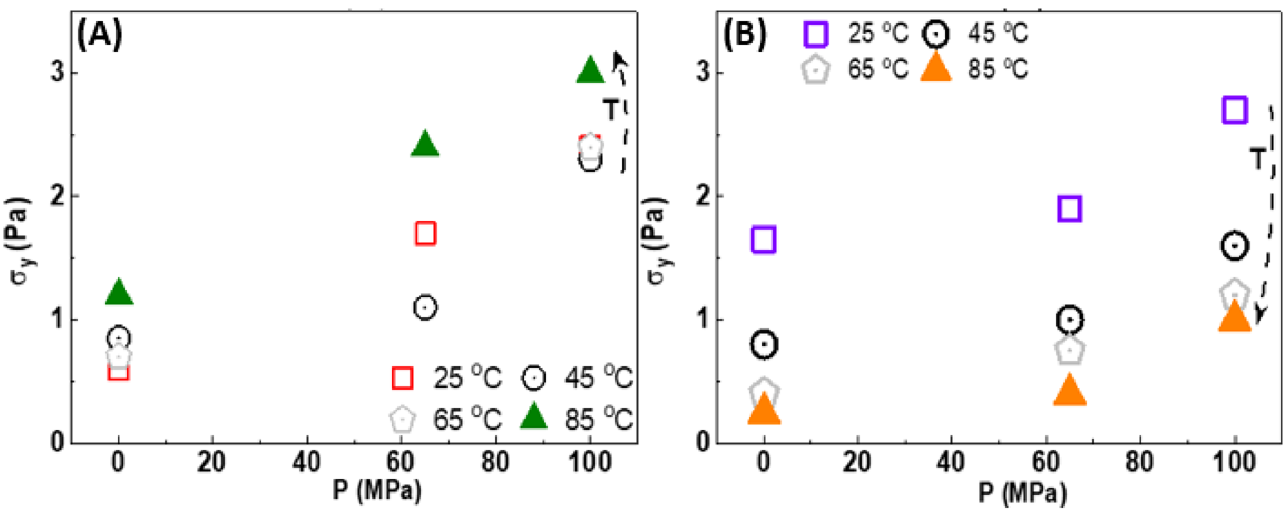

Figure S4. Yield stress, σ_y for a non-aged sample (t_w=0s) as a function of pressure at 25 ℃ (squares), 45 ℃ (circles), 65 ℃ (polygons) and 85 ℃ (triangles) of (A) clay dispersion (5 wt. %) and (B) formulation.

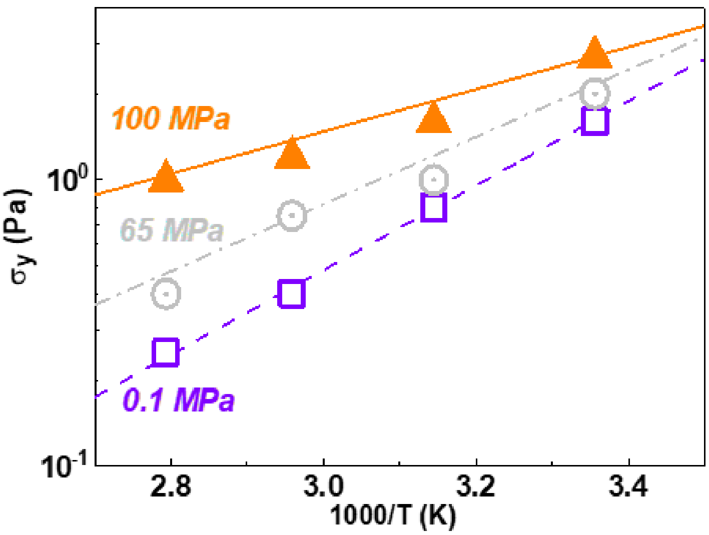

Figure S5. Arrhenius-like representation of yield stress, σ_y as a function of inverse temperature of the formulation at 0.1 (squares) 65 (circles) and 100 *MPa* (triangles).

III. Flow curve at pressure & temperature for aged gels, $t_w > 0$.

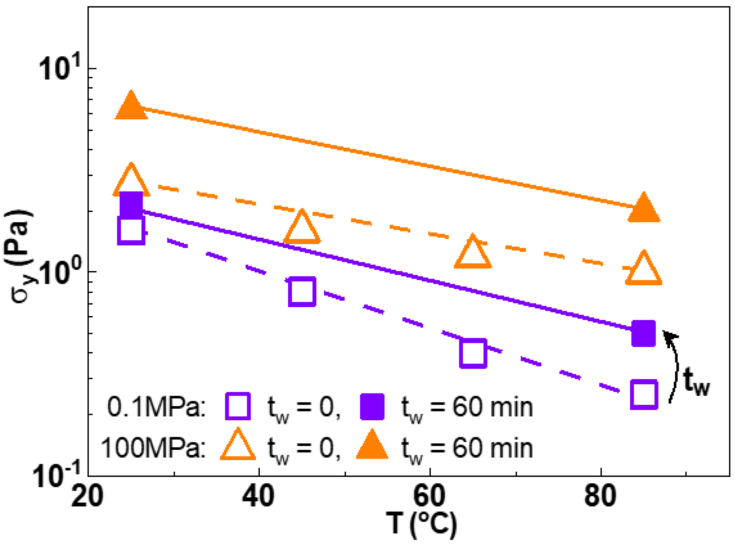

Figure S6. σ_y for a non-aged sample (t_w=0s, open symbols) and for an aged sample (t_w=60 minutes, filled symbols) as a function of: temperature at 0.1 (purple symbols), and 100 MPa (orange symbols) of formulation.

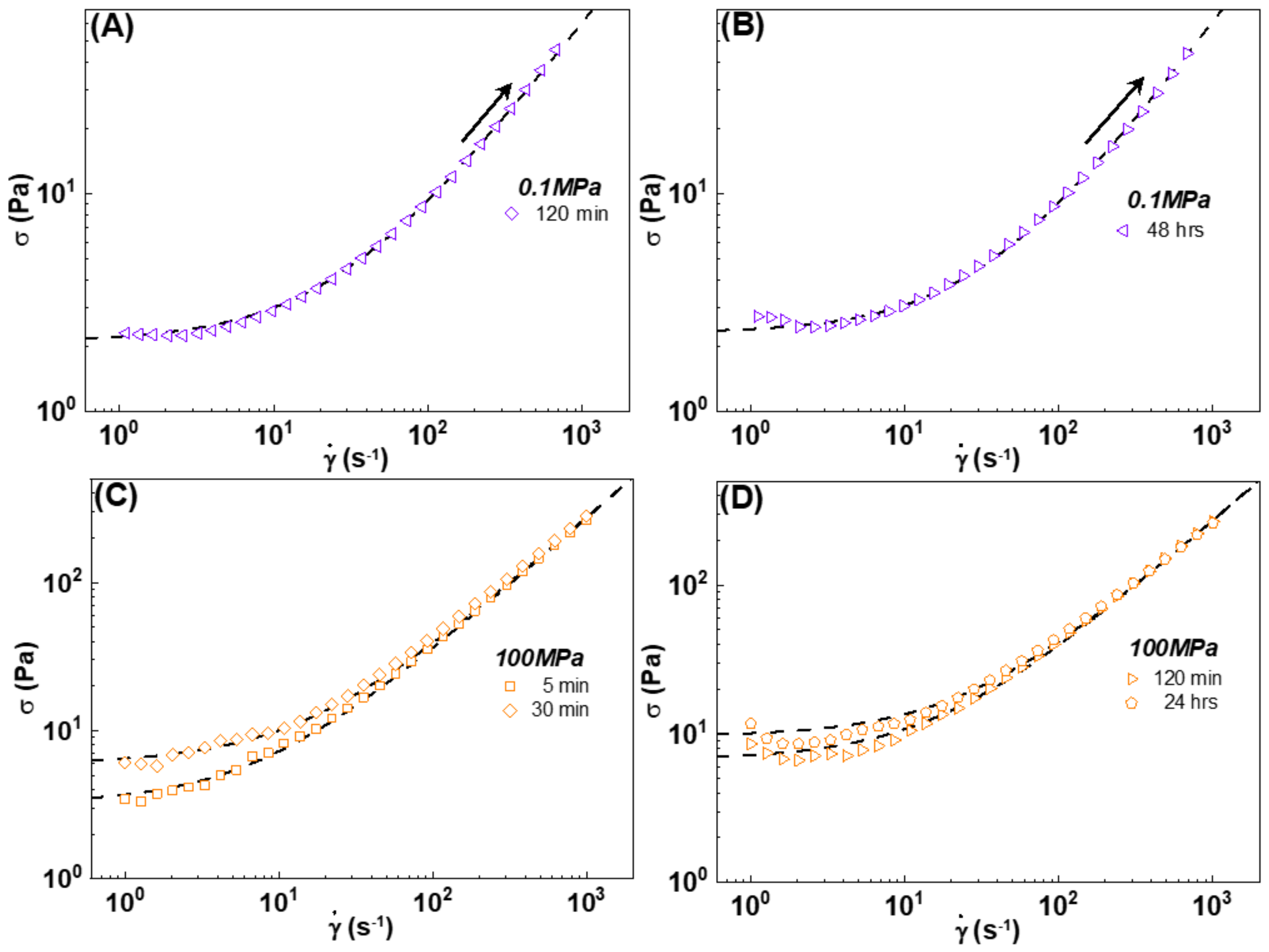

Figure S7. Flow curves of formulation at 0.1 MPa (open purple) at (A) 120 min and (B) 48 hrs and at 100 *MPa* (filled orange symbols) at (C) 5. 30 min and (D) 120 min and 24 hrs. Measurements performed from low to high shear rates (black arrow) after a specific period of waiting time at rest (t_w) as indicated in the legends. Dashed lines represent T-C fits of the experimental data.

IV. Description of Leong Model for yield stress evolution.

The model developed according to Smoluchowski coagulation rate theory[1] implies that the decreasing number of discrete particles (after shear cessation), n, is represented in the simplified form as $\frac{-dn}{dt_w}=k_2 n^2$, where k_2 the coagulation rate constant.[2–4] If we consider that at infinite time after cessation, all the particles are connected, then the concentration of particle bonds is proportional to the total number of individual particles per unit volume, n_0 , and $n_0^{2/3}$, per unit area. In terms of yield stress (proportional to concentration of particle bonds per unit area), the time-evolution of yield stress at

$t_w \to \infty$ can be described as $\sigma_y(t_w) = A\, n_0^{2/3}$ where A a proportionality constant. For any intermediatet_w we should consider the number of the non-interacted particles, n_{t_w}. This returns a number of particle bonds per unit volume $(n¿¿0 - n_{t_w})¿$ and ¿¿, per unit area, respectively. [2] Accordingly, the time-evolution of yield stress at any intermediate time t_w can be described as $\sigma_y(t_w) = A¿$, with the fully broken state, $\sigma_y(t_w=0) = A¿$ where n_E indicate the maximum concentration of non-interacted particles. Substituting the parameters n_0, n_E and A we derive Equation 3.

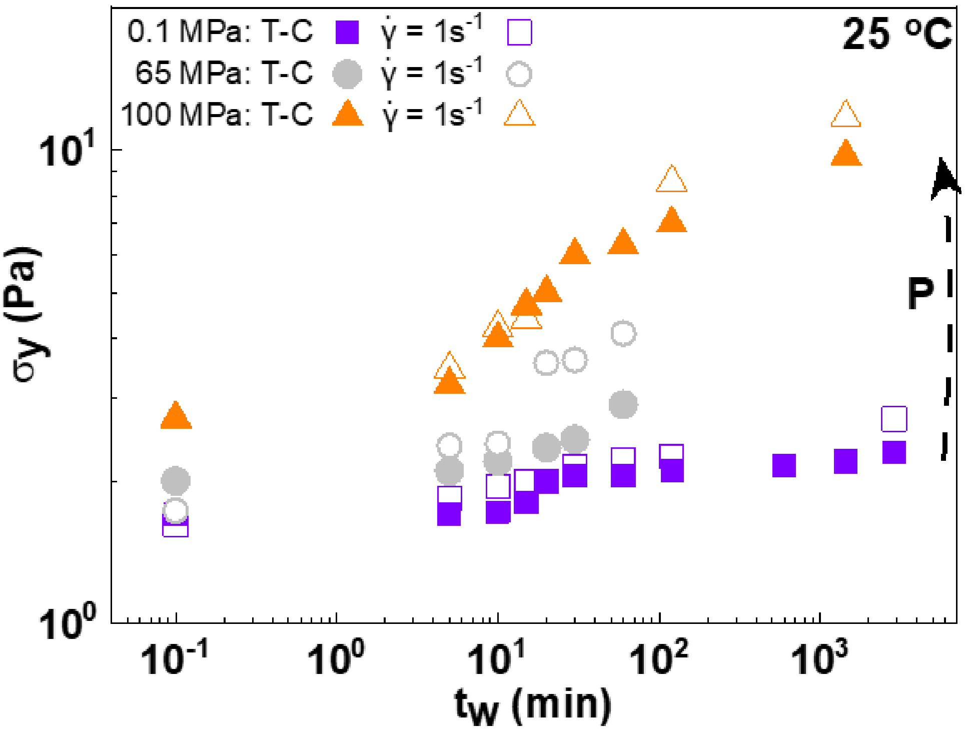

Figure S8. Yield stress, σ_y (Pa) as a function of waiting time, t_w (minutes) of formulation at 25 ℃ at 0.1(purple squares), 65 MPa (grey circles) and 100 MPa (orange triangles) derived from T-C model (filled symbols) and from the first point of the flow curve at $\dot{\gamma} = 1\, s^{-1}$(open symbols).

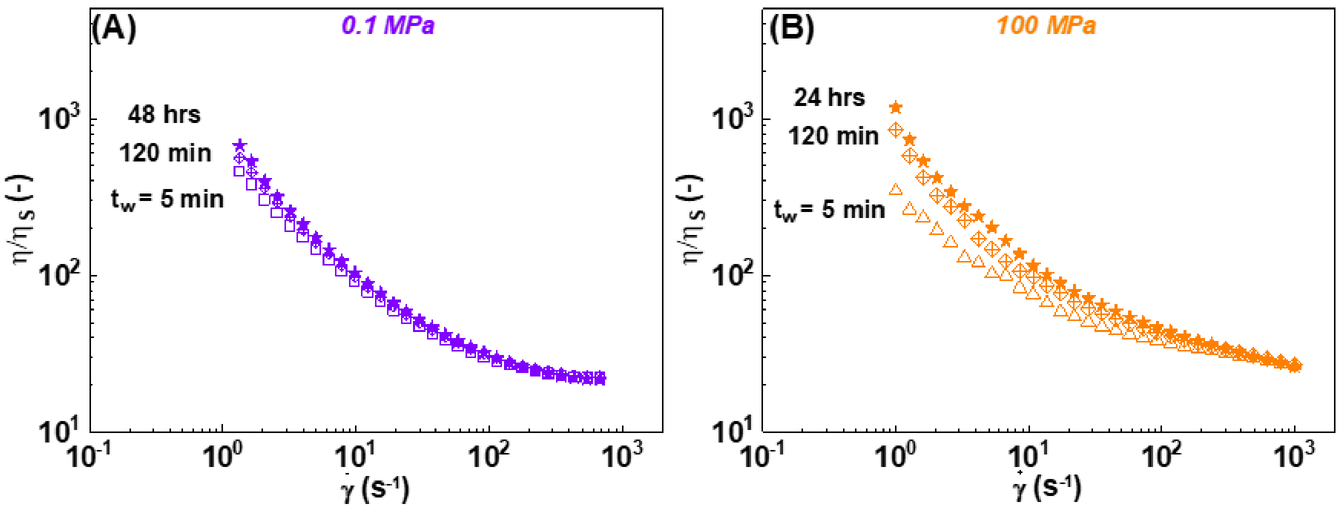

Figure S9. Flow curves (normalized viscosity with solvent viscosity) of the formulation at (A) 0.1 (purple) and (B) 100 *MPa* (orange symbols) at 25 ℃. Measurements are performed from low to high shear rates after different waiting times at rest as indicated in the legends.

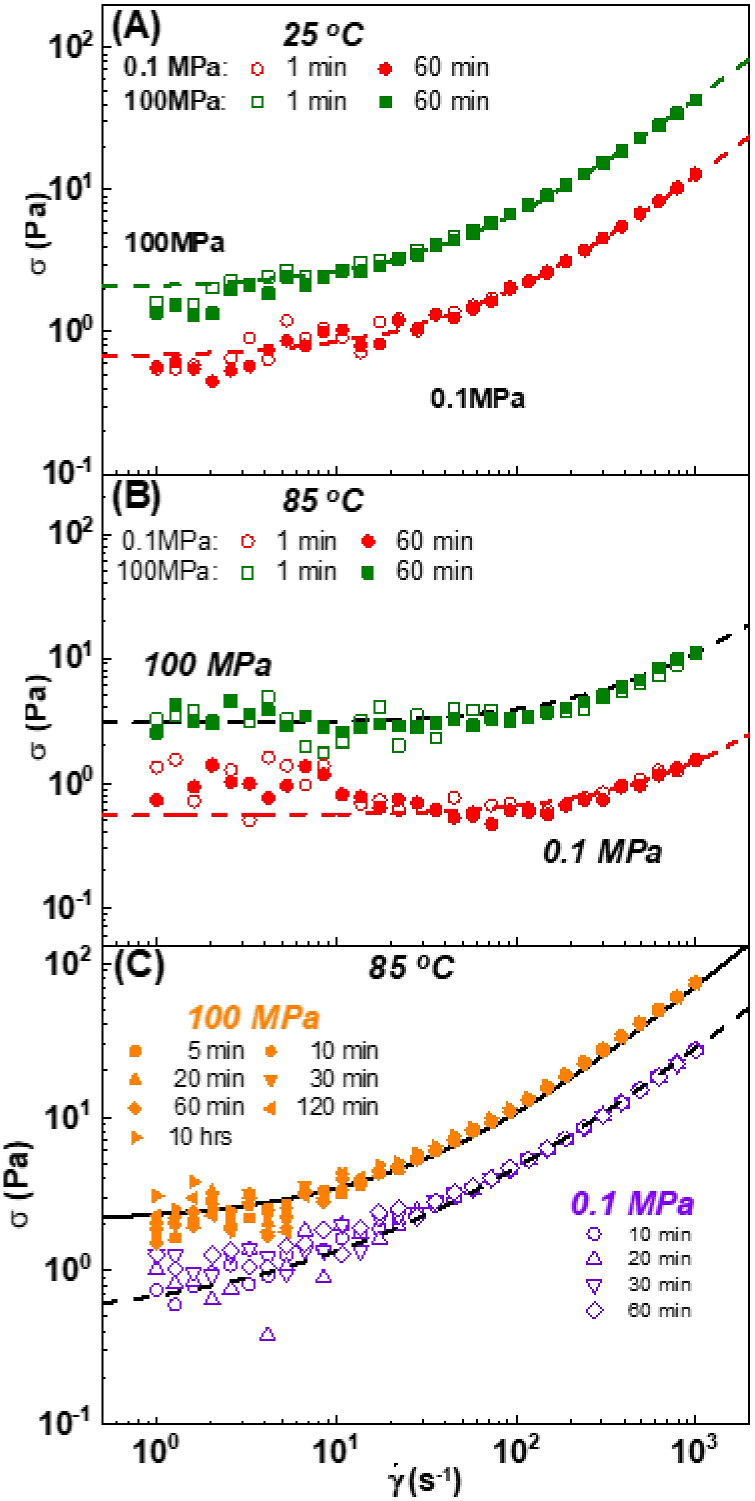

Figure S10. Flow curves of clay dispersions (red symbols) at (A) 25 ℃ and (B) 85 ℃ at 0.1 and 100 *MPa* and (C) formulation at 0.1 (purple symbols) and 100 *MPa* (orange symbols). Measurements performed from low to high shear rates after defined waiting time at rest (t_w) as indicated in the legends. Dashed lines represent T-C fits of the experimental data.

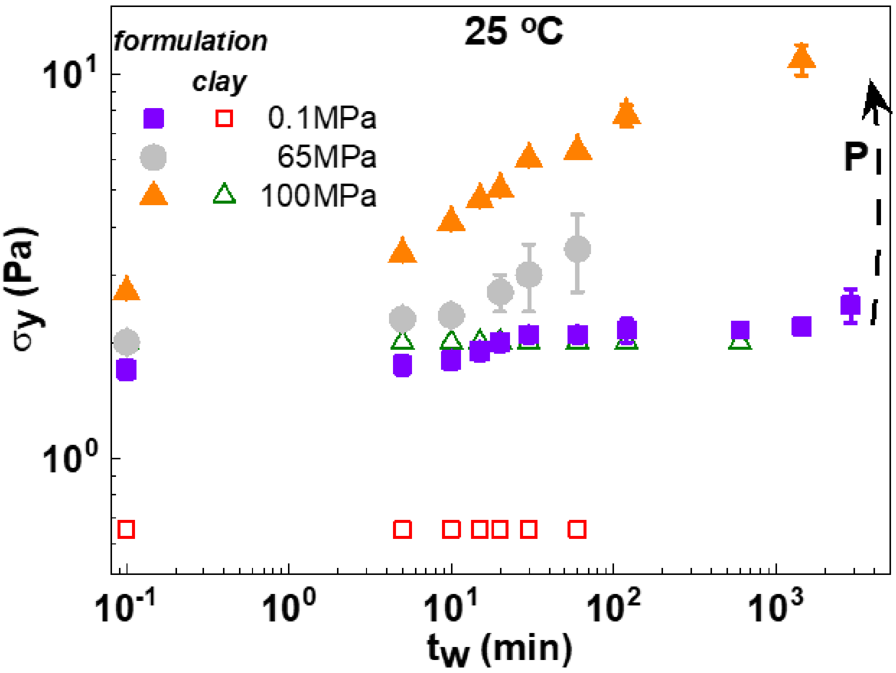

Figure S11. Yield stress, σ_y (Pa) as a function of waiting time, t_w (minutes) of clay dispersions (open symbols) and formulation (filled symbols) at 25 ℃ at 0.1(squares), 65 (circles) and 100 MPa (triangles).

References

(1) Mewis, J. Thixotropy - a General Review. *J. Non-Newton. Fluid Mech.* **1979**, *6* (1), 1–20. https://doi.org/10.1016/0377-0257(79)87001-9.

(2) de Kretser, R. G.; Boger, D. V. A Structural Model for the Time-Dependent Recovery of Mineral Suspensions. *Rheol. Acta* **2001**, *40* (6), 582–590. https://doi.org/10.1007/s003970100180.

(3) Leong, Y. K.; Clode, P. L. Time-Dependent Clay Gels: Stepdown Shear Rate Behavior, Microstructure, Ageing, and Phase State Ambiguity. *Phys. Fluids* **2023**, *35* (12), 123329. https://doi.org/10.1063/5.0167806.

(4) Hattori, K.; Izumi, K. A Rheological Expression of Coagulation Rate Theory. *J. Dispers. Sci. Technol.* **1982**, *3* (2), 129–145. https://doi.org/10.1080/01932698208943630.
